# Nanotextured titanium inhibits bacterial activity and supports cell growth on 2D and 3D substrate: A co-culture study

**DOI:** 10.1016/j.bioadv.2024.213766

**Published:** 2024-01-12

**Authors:** Mohd I. Ishak, Rosalia Cuahtecontzi Delint, Xiayi Liu, Wei Xu, Penelope M. Tsimbouri, Angela H. Nobbs, Matthew J. Dalby, Bo Su

**Affiliations:** a Bristol Dental School, University of Bristol, Lower Maudlin Street, Bristol BS1 2LY, UK; b School of Chemistry, University of Bristol, Cantock’s Close, Bristol BS8 1TS, UK; c Centre for the Cellular Microenvironment, School of Biomedical Sciences, University of Glasgow, Glasgow G12 8QQ, UK; d National Engineering Research Center for Advanced Rolling and Intelligent Manufacturing, Institute of Engineering Technology, University of Science and Technology Beijing, Beijing 100083, China

**Keywords:** 2D and 3D substrate, Co-culture, Nanostructures, Antibacterial, Biocompatible

## Abstract

Medical implant-associated infections pose a significant challenge to modern medicine, with aseptic loosening and bacterial infiltration being the primary causes of implant failure. While nanostructured surfaces have demonstrated promising antibacterial properties, the translation of their efficacy from 2D to 3D substrates remains a challenge. Here, we used scalable alkaline etching to fabricate nanospike and nanonetwork topologies on 2D and laser powder-bed fusion printed 3D titanium. The fabricated surfaces were compared with regard to their antibacterial properties against *Staphylococcus aureus, Escherichia coli*, and *Pseudomonas aeruginosa*, and mesenchymal stromal cell responses with and without the presence of bacteria. Finite elemental analysis assessed the mechanical properties and permeability of the 3D substrate. Our findings suggest that 3D nanostructured surfaces have potential to both prevent implant infections and allow host cell integration. This work represents a significant step towards developing effective and scalable fabrication methods on 3D substrates with consistent and reproducible antibacterial activity, with important implications for the future of medical implant technology.

## 1 Introduction

Medical devices are widely used in modern medicine, with a range of simple devices such as contact lenses, catheters, and blood bags, as well as more complex systems, including heart stents, pacemakers and deep brain neurostimulators [1]. It is estimated that every individual in modern society will undergo a medical implant procedure at least once in their lifetime [2]. In the next few decades, the growing ageing population and increasing rates of human organ failure due to increased ageing and poor lifestyle choices are expected to fuel the demand for medical devices [3,4].

Titanium and its alloys are commonly used in medical procedures such as orthopaedic trauma surgery, total hip and knee arthroplasties, and dental implants. Despite improvements in surgical techniques and hygiene practices, implant-associated infections remain a significant complication and cause of implant failure [5]. These failures are often attributed to aseptic loosening resulting from poor osseointegration or bacterial infection resulting from bacterial infiltration and biofilm formation on the device. Gram-positive bacterial species are the predominant cause of implant-associated infections, with *Staphylococcus aureus* responsible for 30 % of cardiac device infections and 30–40 % of prosthetic joint infections [6]. The combined incidence of infections caused by *Staphylococcus epidermidis, Pseudomonas aeruginosa* and *Enterococcus faecalis* accounts for >75 % of orthopaedic implant-associated infections [6].

Given the growing clinical need for anti-infective medical implants, it is imperative that researchers focus on sustainable and scalable manufacture techniques for clinical application. Nanostructured surfaces, such as nanowires, nanospikes, nanopillars and nanocones, have potential to prevent bacterial infection of medically relevant materials including titanium, polymers and ceramics [4,7–10]. However, their clinical efficacy remains untested, and their approval for commercial or clinical use remains unclear. This is likely due, in part, to difficulties in translating nanostructured surfaces from 2D to 3D substrates, which are more practical for medical applications. Therefore, developing effective and scalable fabrication methods for nanostructures on 3D substrates with reproducible antibacterial activity is a key challenge for the field.

In our previous study, we successfully fabricated 3D porous titanium scaffolds using selective laser melting (SLM) [11]. It was found that a hybridised primitive-gyroid structure with 60 % porosity (PG60) had the best permeability, excellent cytocompatibility, and mechanical properties that were comparable to cortical bone. These properties made it a promising candidate for bone implant material design. In this study, we first employed a sustainable and scalable alkaline etching technique to fabricate two distinct nanostructured surfaces on 2D substrates of pure titanium. We thoroughly characterised and tested the antibacterial properties of and stem cell response to the fabricated surfaces. We then successfully replicated the nanostructures on SLM-fabricated commercially pure titanium (cpTi) 3D substrates using the same etching technique and evaluated their cytocompatibility and antibacterial efficacy. To assess the mechanical properties and permeability of the 3D substrate, we conducted finite elemental analysis (FEA) and supported the results through experimental validation. Additionally, we examined the cytocompatibility and antibacterial efficacy of the nanostructures against both Gram-negative and Gram-positive bacteria.

## 2 Experimental

### 2.1 2D substrate surface preparation

**Scheme 1:**
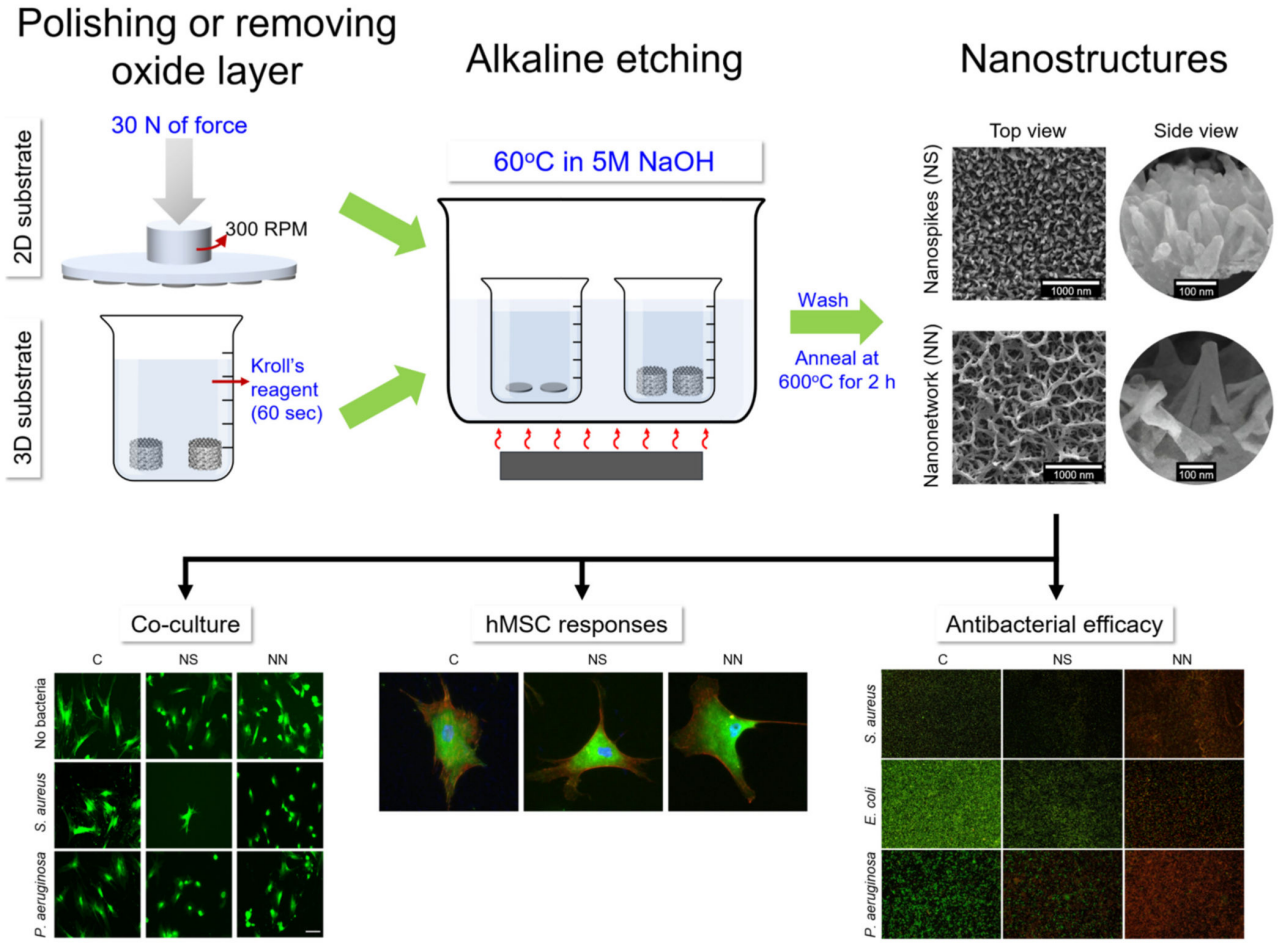
Preparation of NS and NN on 2D and 3D cpTi substrates and their subsequent antibacterial efficacy, cell responses, and co-culture experiments.

Titanium discs (Ø = 11 mm, Grade 1) were polished to grit levels of 4000 using Struers tegraPol-15. The discs were then cleaned by sonication (Grant XUB5) for 15 min in deionised water and immersed in absolute ethanol (Merck) for 10 min before being blow-dried with compressed air. The discs were placed in an upright position using a custom-made PTFE holder and immersed in a beaker containing pre-warmed 5 M NaOH (Fischer) solution at 60 °C. The nanospike (NS) surface was generated by etching the cpTi discs for 1 h while the nanonetwork (NN) was etched for 16 h. After the etching process, the discs were washed thoroughly using dH_2_O and ethanol (Merck) before being left to dry overnight. The final step involved placing the discs in the chamber furnace for calcination for 2 h at 600 °C with a heating rate of 10 °C/min. The fabrication process is illustrated in Scheme 1. The discs were cooled and stored in a sterile, enclosed plastic Petri dish until use.

### 2.2 3D substrate surface preparation

#### 2.2.1 Manufacturing of TPMS-based graded scaffolds

Graded scaffolds were fabricated by an SLM machine (SLM 125HL, SLM Solution). The layer thickness, laser power, scanning speed, and hatching distance were 30 μm, 150 W, 385 mm/s, 0.12 mm, respectively. After manufacturing, graded scaffolds were removed from the baseplate by wire cutting, and then were cleaned ultrasonically for 30 mins. The fabricated samples had a unit cell size of 2 mm, cell thickness of 0.26 mm, diameter of 8 mm, height of 6.5 mm, and porosity of 60 %.

#### 2.2.2 Alkaline etching on 3D substrate

The cpTi 3D (3 DC) substrate was etched using Kroll’s reagent (100 mL dH_2_O, 2 mL HF 40 wt% (Fischer), 4 mL HNO_3_ 65 % wt% (Merck)) for 60s to remove the oxide layer on the surface. After the acid etching process, the substrate was thoroughly rinsed with water and ethanol before being allowed to air dry in a fume hood. The NS and NN structures were then fabricated on the 3D substrate using the same parameters as those used for the 2D substrate. Briefly, the substrate was placed in a beaker containing pre-warmed 5 M NaOH at 60 °C. The 3D substrate was etched for 2 h to make the nanospikes (3DNS) and 16 h for the nanonetwork (3DNN). The substrates were washed extensively with dH_2_O and ethanol for 10 min, air dried, and stored in airtight containers for further experimentation.

### 2.3 Simulation and mechanical characterisation of 3D substrate

#### 2.3.1 Finite elemental analysis (FEA) of the 3D substrate

Statics mechanics and permeability simulation were performed by FEA. In the simulation of statics mechanics, the graded scaffold materials were set as Ti, and the top and bottom rigid plates were applied with a vertical force of 120 N and fixed, respectively. For permeability simulation, water was selected as a liquid and simulated permeability was calculated by the following equation:

(1) $$k=\frac{\nu •\mu •L}{\Delta P}$$

where *k* is the permeability coefficient, m^2^; *v* is the fluid velocity, m/s (0.001 m/s in this study to ensure laminar); the dynamic viscosity coefficient of fluid *μ* is 10^-3^ Pa⋅s; *L* is the height of the porous structure, m (0.01 m); Δ*P* is the pressure drop between the inlet and outlet, Pa, and the pressure of outlet is set as 0.

#### 2.3.2 Microstructures, mechanical properties, and permeabilities characterisation

Microstructures were observed by SEM. Compressive mechanical property was tested by a universal testing machine at room temperature. The strain rate is 0.01 mm/s according to ISO 13314: 2011(E). The permeabilities were tested by the falling head method. The detailed test process is described in our previous studies [12].

#### 2.3.3 Total surface area and volume estimation

The total surface area and volume of the 3D substrate were estimated using morphometric analysis of the 3D models as previously described [13]. Briefly, the same 3D models that were used for the substrate fabrication were imported in Blender (V3.4.0). NeuroMorph add-on was applied on the 3D model to measure both the total surface area and volume of G60 and G50 substrate.

### 2.4 Bacterial strains and culture conditions

*Escherichia coli* strain K12 [14], *P. aeruginosa* strain ATCC 27853 (ATCC) and *Staphylococcus aureus* strain Newman [15] were used in this study (Table S1). The strains were selected to represent a range of bacteria with different properties, i.e. Gram-type, cell morphology and motility. Bacteria were cultured overnight (16 h) at 37 °C, 220 rpm in Mueller Hinton broth (Sigma-Aldrich). For use in assays, bacteria were subcultured to an OD_600_ 0.1 and grown to mid-exponential phase. Mid-exponential phase cultures were preferred to guarantee that surfaces could be reproducibly inoculated with high numbers of viable, metabolically active bacteria.

### 2.5 Antibacterial efficacy of nanostructured surfaces

#### 2.5.1 BacTiter-Glo

Sterile cpTi 2D samples were placed inside white 24-well plates (PerkinElmer, MA, USA) and inoculated with 400 *μ*L (10^6–7^ CFU) of bacterial suspension. For the 3D substrate, samples were placed inside 48-well plates and inoculated with the same number of bacteria. Plates were incubated at 37 °C for 3 h or 24 h under static conditions, after which time 30 *μ*L of bacterial suspension was removed from each well and mixed with 30 *μ*L of BacTiter-Glo™ (BTG) assay reagent (Promega) in a new, white 24-well plate to allow quantification of non-adherent viable cells. The test surfaces were washed gently with Tris-HCl buffer (pH 7.0) and, for the 2D samples, were transferred to a new, white 24-well plate, where 30 *μ*L of BacTiter-Glo™ reagent was then applied to each surface to allow quantification of adherent viable cells. For the 3D substrate, 400 *μ*L of BTG reagent was added to each well of the 48-well plate for 5 min before the 3D samples were transferred to white 24-well plate. In both instances, plates were incubated for 5 min in the dark prior to relative luminescence unit (RLU) measurement using a Tecan Infinite F200 PRO microplate reader with automatic attenuation and an integration time of 1000 ms and settling time of 0.15 ms. The values of RLU were converted to CFU using standard curve specific for each strain of bacteria (Suppl. Fig. S2).

#### 2.5.2 Scanning electron microscopy (SEM) sample preparation

Briefly, bacteria adhered to the surface were fixed overnight at 4 °C with 2.5 % glutaraldehyde (EM grade, Sigma Aldrich) in 0.1 M sodium cacodylate (98 % pure, Acros Organics). Then, samples were progressively dehydrated with 20 %, 40 %, 60 %, 80 % and 100 % ethanol (Sigma-Aldrich) for 10 min each before drying using a critical point dryer (Leica CPD300). The dried samples were mounted onto 0.5-in. stubs (Agar Scientific) and sputter coated with gold and palladium for SEM.

#### 2.5.3 Bacterial LIVE/DEAD staining

Following the incubation with the nanostructured surfaces (following Section 2.5.1 before the addition of BTG reagent), 400 *μ*L of diluted LIVE/DEAD stain (Thermo L3224) (3 *μ*L stain in 1 mL of Tris-HCl) was added to each test surface and incubated for 15 min in the dark at room temperature. Samples were then washed with Tris-HCl buffer to remove excess stain and visualised by fluorescence microscopy.

### 2.6 Atomic force microscopy (AFM)

**Fig. 1:**
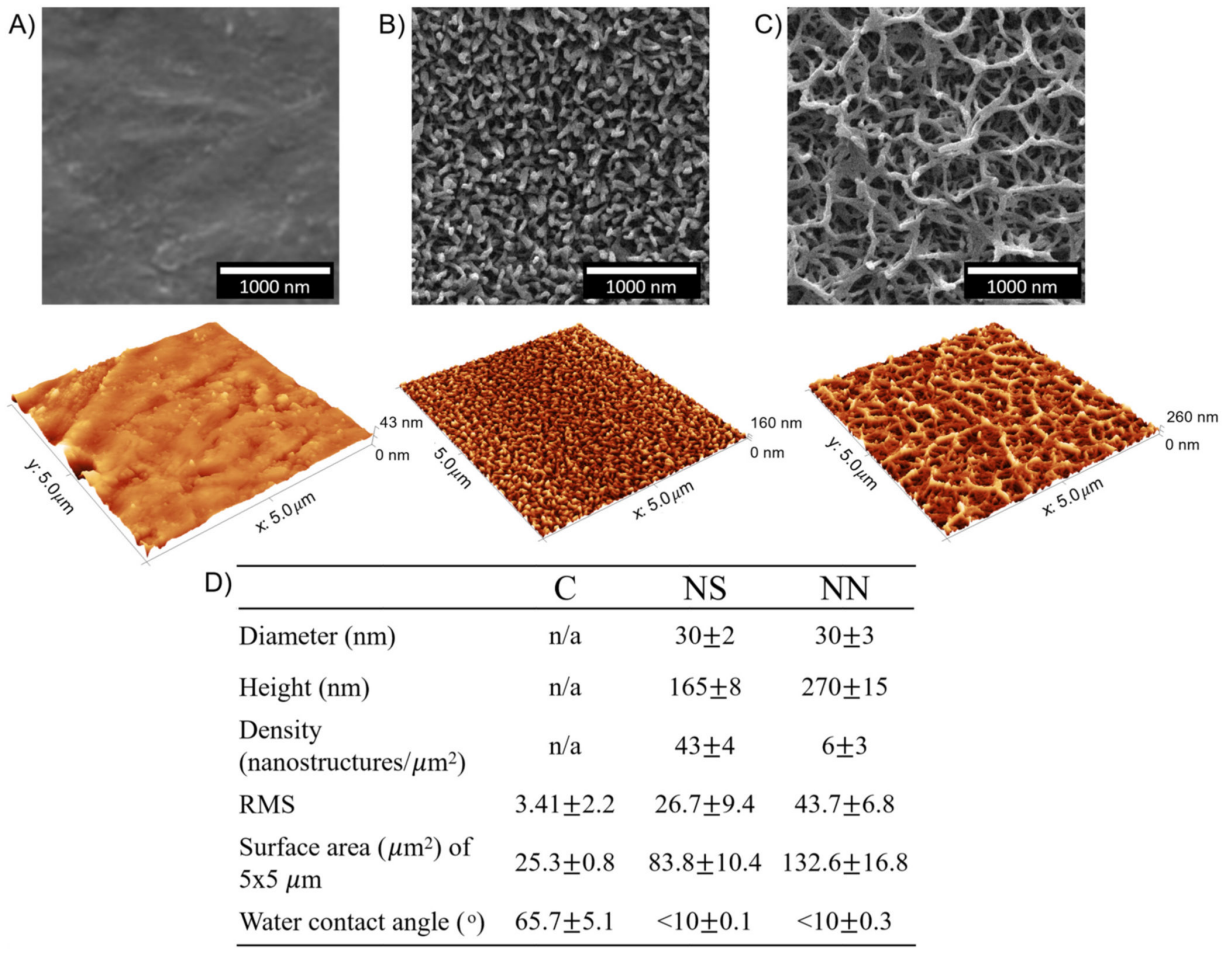
Representative images of nanotopographies from SEM and AFM. SEM (top panel) and AFM images (bottom panel) of TiO_2_ nanostructures of (a) control surface, (b) nanospikes (NS) and (C) nanonetwork (NN). (D) Summary table of quantified topography, roughness, and water contact angle of the surfaces.

Nanospikes were analysed using AFM and the average height, surface area and RMS (average-root mean square) roughness were quantitatively measured. Nanospikes were imaged using an AFM (Digital Instruments INC Nanoscope IIIa Atomic Force Microscope) and contact probe tips (MikroMash). An area of 25 μm^2^ with a scan rate of 1.0 Hz and 512 lines was measured. The average roughness (*R*_a_), maximum peak-to-valley heights (*R*_max_), and surface area were quantified using Gwyddion v2.56 (Fig. 1).

### 2.7 Contact angle measurement

42TThe contact angle of the flat control, NS, and NN surfaces were measured using a sessile drop method with a drop42T shape analyser (DSA100, KRÜSS). The system was first calibrated, and the surface tension of the MilliQ water was measured. A 2 *μL* droplet of MilliQ water was deposited on the substrate, and the resulting static contact angle *θ* was recorded over 30 s after the droplet was deposited.

### 2.8 Stem cell culture

Stro 1^+^ human mesenchymal stromal cells (hMSCs) were isolated and donated by Prof. Richard Oreffo from the University of Southampton. The cells were cultured using high glucose DMEM (Sigma, D5671) containing NaHCO_3_, supplemented with 1 % antibiotics (100 units mL^-1^ penicillin, 100 μg mL streptomycin), 2 mM GlutaMAX supplement (Invitrogen, USA), 10 % *v*/v foetal bovine serum (FBS), and 1 % non-essential amino acids (11140050). Cells were used at passage 2 or 3.

#### 2.8.1 Co-culture media and bacterial inoculum

The conditions for cultivating hMSCs on the nanotextured surfaces were taken from previously published work [16]. Low serum (1 % FBS) was used, as this is the minimal quantity that can sustain hMSC viability. Combined with 0.3 % penicillin/streptomycin, these conditions sufficiently slowed the growth of bacteria to allow co-culture with hMSCs to be maintained for 24 h. The bacterial inoculum of 10^3^ CFU for the co-culture system was selected as the lowest bacterial density at which effects of the nanotextured surfaces were recorded. 10,000 hMSCs were seeded in low serum in each surface and left to adhere overnight. The next day, the media was replaced with 1 mL of low serum media (1 % FBS and 0.3 % pen/strep) containing 10^3^ CFU and co-cultured for 24 h.

### 2.9 hMSCs LIVE/DEAD staining

Before seeding with hMSCs, the titanium discs were washed by soaking in ethanol for at least 1 h. After drying, the discs were further sterilised by irradiating with UV light for 30 min on each side.

hMSCs were seeded onto Ti 2D discs in a 24-well plate (10,000 cells per disc) in seeding media (growth medium with all supplements, 2 % FBS, 0.3 % pen/strep) to ensure cell attachment overnight. The next day, the discs were rinsed twice with PBS and 0.5 mL of a serum-free DMEM solution containing 0.5 μM calcein AM and 2 μM ethidium homodimer-

1. The cells were incubated for 15 min in the dark and then imaged in PBS using an inverted fluorescence microscope (EVOS M7000). Cell quantification was performed using ImageJ.

### 2.10 Focal adhesions

#### 2.10.1 Immunostaining

hMSCs were seeded onto Ti 2D discs in a 24-well plate (10,000 cells per disc) in seeding media. After 24 h, the cells were rinsed and fixed with 4 % paraformaldehyde in PBS for 15 min at 37 °C. Then, the membrane was permeabilised using 0.05 % Triton X-100 (Sigma) in PBS for 5 min at 4 °C. The samples were blocked using 1 % BSA in PBS for 10 min with gentle agitation. Primary antibody vinculin (VLN01, 1:150 dilution) and Rhodamine Phalloidin (R415, 1:500 dilution) were added in 1 % BSA/PBS buffer overnight at 4 °C. After rinsing the discs with 0.5 % Tween-20/PBS 5 times for 5 min, secondary antibody (anti-mouse IgG (H + L) biotinylated BA-2000, 1:100 dilution) was added in 1 % BSA/PBS buffer for 1 h at 37 °C. The samples were washed as before, and streptavidin fluorescein was added as the fluorescent probe (streptavidin fluorescein SA-5001, 1:100 dilution) and incubated for 30 min at 4 °C. Excess probe was removed by washing the samples, as previously stated, and samples were then mounted using Vectashield-DAPI on a glass slide, before being imaged using a fluorescence microscope (EVOS M7000).

#### 2.10.2 In-cell Western blot

hMSCs were seeded onto Ti 2D discs in a 24-well plate, fixed with 4 % paraformaldehyde, and permeabilised using 0.05 % Triton X-100, as previously described. The samples were blocked using 1 % milk in PBS for 1.5 h. Primary antibody (FAK H1 sc-1688 and pFAK Y397 D20B1 at 1:100 dilution) was added overnight. The next day, samples were washed five times for five minutes using 0.1 % Tween-20 in PBS, then secondary antibody goat anti-mouse (926–32,210) and goat anti-rabbit (926–68,071) were added, respectively, at 1:800 dilution for 1 h at room temperature in the dark. Samples were washed again and left to air dry for at least 48 h at 4 °C in the dark before analysing using Licor Odyssey M.

### 2.11 3D hMSC culture

hMSCs were seeded at 20,000 cells per surface for 1 h at 30 RPM in seeding media. A higher inoculum was used due to the larger surface area of the 3D samples compared to the 2D samples. Then, samples were placed into a 48-well plate for further incubation for 24 h at 37 °C, before Alamar Blue (Invitrogen DAL1025) was used according to the manufacturer’s instructions to determine cell viability. Briefly, 50 μL of Alamar Blue in 450 μL of DMEM was added to each sample and 100 μL aliquots in duplicate were collected and analysed using a plate reader (Multiskan FC, Thermo Scientific) at 570 nm. A standard curve was used to determine cell attachment and viability.

### 2.12 hMSC/bacteria co-culture

First, hMSCs were seeded at 10,000 cells per Ti surface in a 24-well plate overnight in seeding media. At the same time, overnight broth cultures were set-up for *P. aeruginosa* (DMEM without antibiotics) or *S. aureus* (Mueller Hinton). The next day, bacteria were sub-cultured, grown to log phase (OD_600_ 0.3 for *P. aeruginosa*, OD_600_ 0.5 for *S. aureus*) and 1 mL (10^3^ CFU) of bacterial suspension was added to each Ti surface in co-culture media (growth medium with all supplements, 1 % FBS, 0.3 % pen/strep) and incubated overnight at 37 °C.

For SEM preparation, Ti discs were then carefully washed once with PBS and fixed using 2.5 % glutaraldehyde overnight at 4 °C. To improve the contrast, the samples were rinsed in 0.1 M sodium cacodylate and treated with 1 % osmium tetroxide for 1 h at room temperature and then washed 3 times using distilled water for 10 min. This was followed by 0.5 % uranyl acetate staining for 1 h in the dark. An ethanol dehydration series was performed (30–100 %) and a hexamethyldisilane step was conducted prior to sputter coating (20 nm gold/palladium). Samples were imaged on a JEOL IT100 SEM, using 10 Kv on Secondary Electron Detector (SED) mode. TIFF images were captured using JEOL Intouch Scope software version 1.03.

### 2.13 Statistical analysis

All statistical analyses were performed using Microsoft Excel (Microsoft 365) and GraphPad Prism V9. Data were analysed by ANOVA with Tukey HSD post-hoc test and *p-*values of <0.05 were considered significant. Unless otherwise stated, data are representative of three experimental replicates (*n* = 3) performed in duplicate.

## 3 Results

### 3.1 Surface characterisation of nanostructured 2D Ti substrate

The nanotopographies of flat control and two nanostructured TiO_2_ surfaces were characterised using AFM and SEM. The diameter and density were quantified using SEM and analysed using FIJI (ImageJ, Particle Analysis). The RMS and surface area were quantified using AFM and analysed with Gwyddion V2.62. The height was quantified by AFM and confirmed by SEM from the cross-section analysis. In general, TiO_2_ nanostructures generated in this study had the same tip diameter of around 30 nm, regardless of the etching time. However, as the etching time increased (up to 16 h), the nanospike (NS) started to grow taller and formed a nanonetwork (NN). This growth mechanism resulted in much taller nanostructures (270 nm) when etched for 16 h as compared to nanostructures that were etched for 1 h (165 nm), along with the morphology change from NS to NN (Suppl Fig. S1).

Other parameters that were significantly changed due to the growth mechanism (1 h vs. 16 h etching time) were **roughness (RMS)**: 26.7 vs. 43.7, topography **density:** 43 nanostructures/*μ*m^2^ vs. 6 nanostructures/*μ*m^2^, and **surface area:** 83.8 *μ*m^2^ vs. 132.6 *μ*m^2^ (Fig. 1). However, the hydrophobicity of NS and NN was identical, with both superhydrophilic with a contact angle of less than 10° compared to that of the flat cpTi surface at 65.7°.

### 3.2 Mechanical and permeability characterisation of 3D printed Ti substrate

The mechanical and permeability properties of the 3D substrate were characterised using finite elemental analysis (FEA), as described in our previous work [11]. However, this 3D substrate differed from the former study, being a non-hybridised gyroid structure with 60 % porosity. We opted for this design based on findings that suggested it may possess better mechanical characteristics, more akin to those of cortical bone, than our prior design [12].

**Fig. 2:**
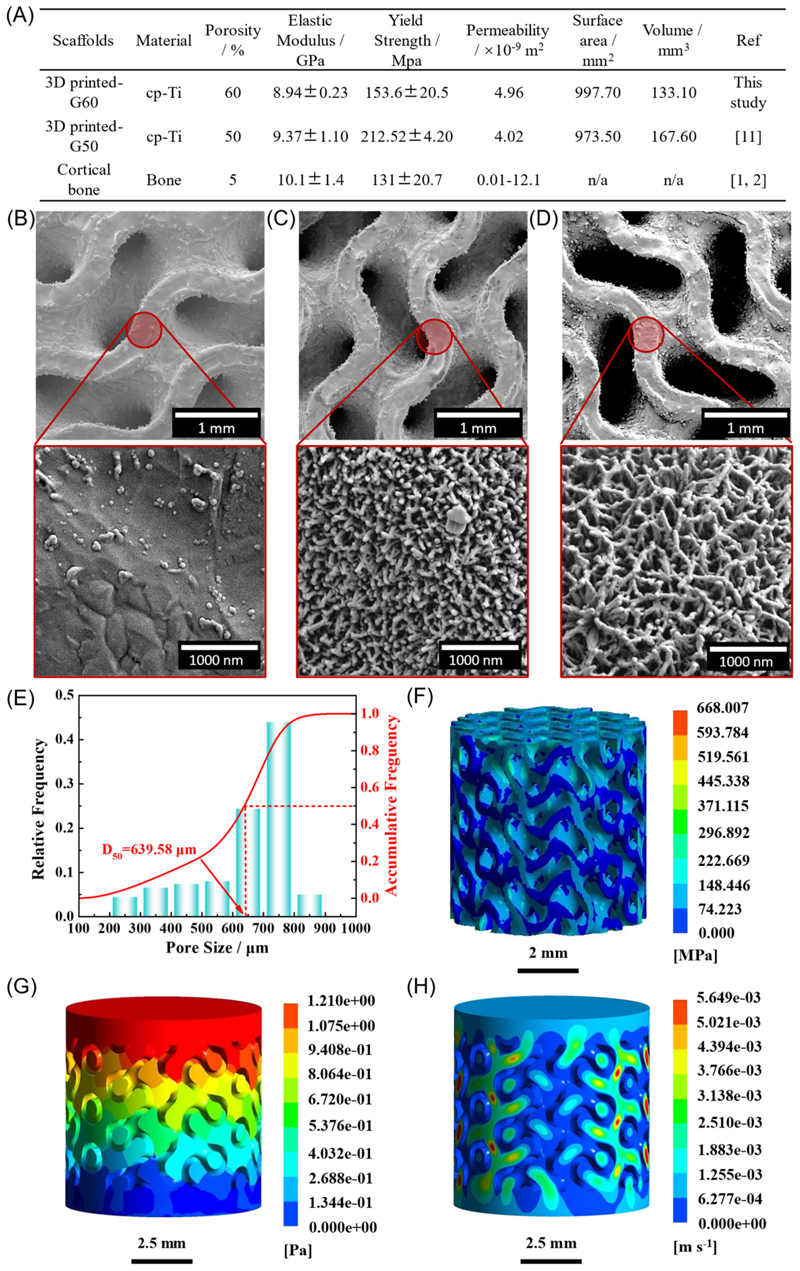
Mechanical properties and permeability simulation study of the 3D cpTi substrate. (A) Mechanical characteristics of the 3D substrate compared to G50 substrate and cortical bone. SEM images of low (top) and high (bottom) magnification of (B) cpTi 3D control (3 DC) substrate, (C) 3D nanospikes (3DNS) substrate, and (D) 3D nanonetworks (3DNN) substrate. FEA analysis of the 3 DC substrate pore size distribution (E), von-Mises stress (F), fluid pressure simulation (G), and fluid velocity distribution (H).

Overall, the mechanical properties of LSM fabricated cpTi substrate with 60 % porosity with a gyroid shape (G60) were found to match the important mechanical properties of cortical bone such as elastic modulus, yield strength, and permeability (Fig. 2A). The G60 substrate was found to have a lower elastic modulus and yield strength when compared to the G50 (50 % porosity) but within the range for other cortical bone mechanical properties. More importantly, the G60 substrate had higher permeability compared to all our previous designs [12]. The SEM images of the etched G60 surface also confirmed that the nanostructures generated on the 3D substrate were identical to those formed on the 2D substrate (Fig. 2B-D). From the FEA study, it was found that the stress distribution was relatively uniform (Fig. 2F) while the fluid pressure was found to drop gradually from the inlet to outlet (Fig. 2G). The flow velocity of the 3D substrate was higher in the center of the channel compared to the surrounding area (Fig. 2H).

### 3.3 Bacterial response to nanostructures on 2D Ti substrate

**Fig. 3:**
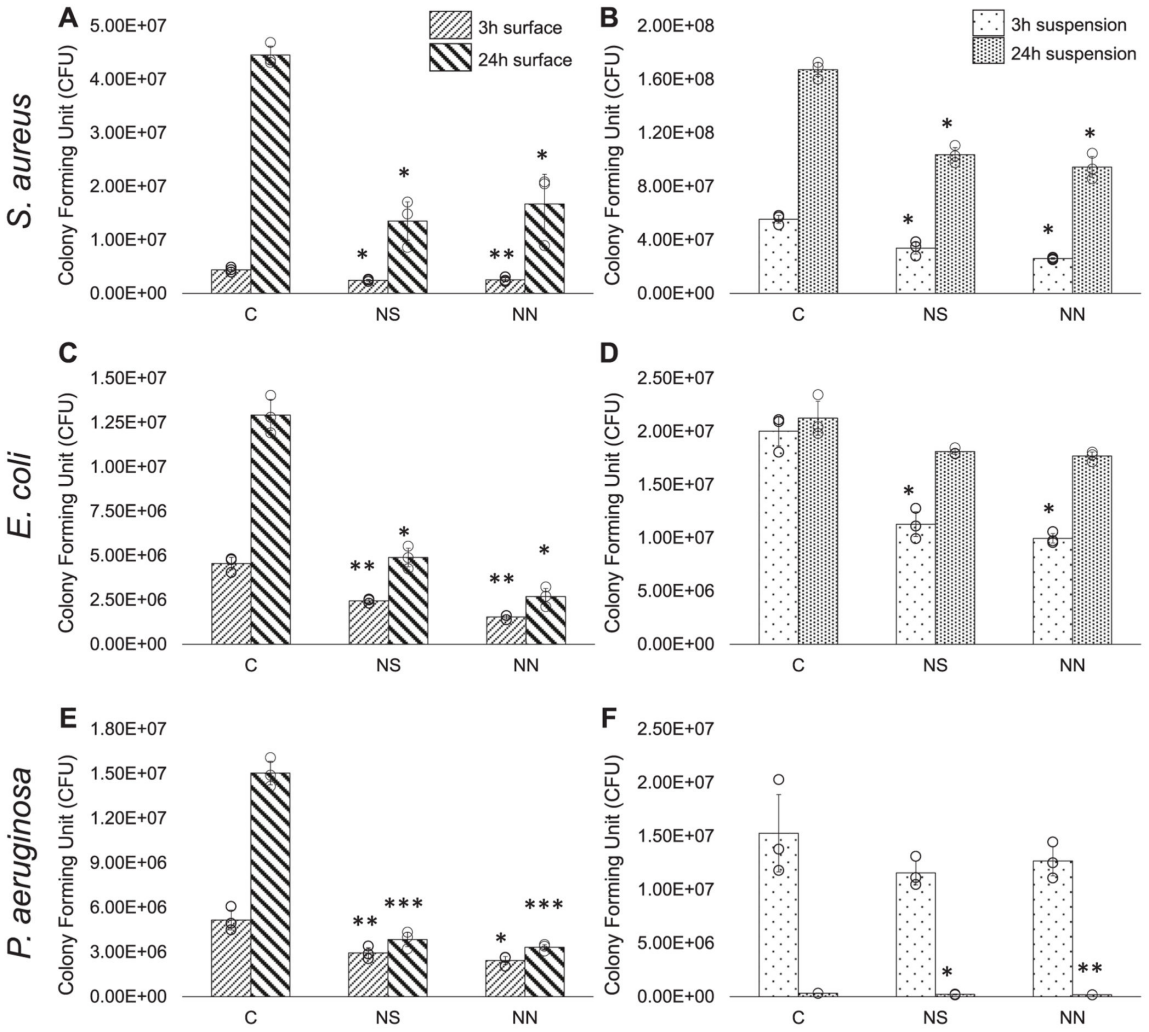
Metabolic activity of bacteria on 2D cpTi surfaces or in the surrounding suspension as measured by BTG assay after 3 h or 24 h incubation. (a,b) *S. aureus*, (c, d) *E. coli* and (e,f) *P. aeruginosa*. C, flat control; NS, nanospike; NN, nanonetwork. Data are presented as mean ± SD. *P < 0.05, **P < 0.01, and ***P < 0.001 compared to control and other surfaces, as determined by one-way ANOVA with Tukey HSD post-hoc test; *n* = 3.

The antibacterial properties of the nanostructures on 2D substrates were quantitatively analysed using a metabolic assay, BacTiter-Glo, and confirmed using LIVE/DEAD stain. After 3 h of static incubation, it was found that all of the tested bacterial strains were significantly affected by both NS and NN when compared to the control. This was observed both for bacteria directly attached to the surfaces (Fig. 3A,C,E) and in planktonic phase in the surrounding suspensions (Fig. 3B,D,F). Overall, the metabolic activity of the adherent *S. aureus* was reduced by 45 % for NS and 42 % for NN compared to flat control (Fig. 3A). Reductions of 46 % and 66 % for *E. coli* and of 43 % and 53 % for *P. aeruginosa* on NS and NN surfaces, respectively, were also recorded (Fig. 3C,E).

When the incubation time was extended to 24 h, bacterial proliferation on the flat control surface was clearly evident. Compared to after 3 h, numbers of viable *S. aureus* reached 4.5 × 10^7^ CFU (904 % increase) (Fig. 3A), while *E. coli* and *P. aeruginosa* reached 1.3 × 10^7^ CFU (184 % increase) and 1.5 × 10^7^ CFU (192 % increase), respectively (Fig. 3C,E). By contrast, the change in cell numbers on both nanostructured surfaces from 3 to 24 h was not significant for the two Gram-negative bacterial species (Fig. 3C,E). Proliferation of *S. aureus* was diminished on the nanostructured surfaces relative to flat control but cell numbers still increased by 450 % and 551 % on the NS and NN surfaces, respectively (Fig. 3A).

For *S. aureus* and *E. coli* in the surrounding suspensions, differences in metabolic activity after 3-h incubation across the surfaces were similar but less pronounced compared to those of bacteria bound directly (Fig. 3B,D). For *P. aeruginosa*, no significant differences in metabolic activity were seen across the surface types (Fig. 3F). After 24-h incubation, proliferation in the suspension was seen for *S. aureus* across all three surfaces compared to at 3 h. Nonetheless, viable cell numbers were significantly diminished on the nanostructured surfaces relative to flat control (Fig. 3B). Reduced viability on NS and NN surfaces compared to control was also seen for *P. aeruginosa* after 24 h although in this case, metabolic activity overall was lower than at 3 h (Fig. 3E). Proliferation of *E. coli* from 3 to 24 h was evident but at 24 h, levels of viability were comparable across the 3 surface types (Fig. 3D).

**Fig. 4:**
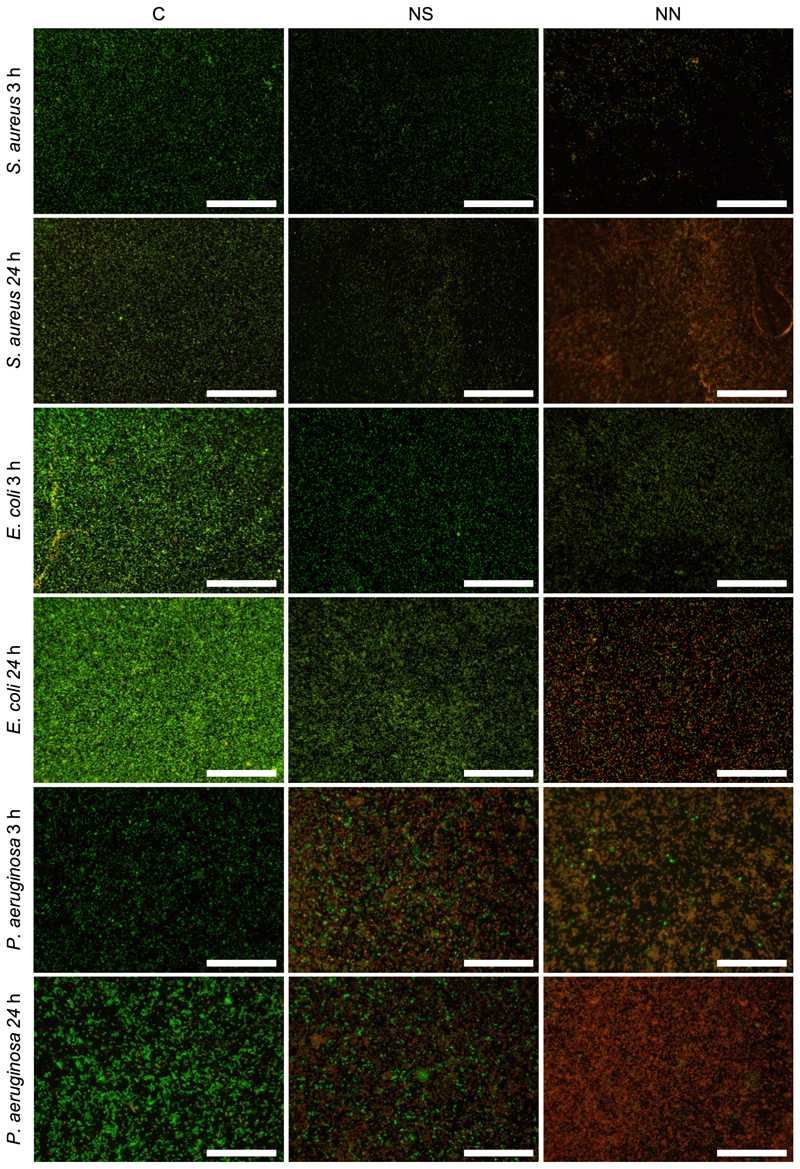
Representative fluorescence micrographs of bacteria attached to 2D cpTi surfaces after 3 h or 24 h incubation. Bacterial suspensions of *S. aureus* (top rows), *E. coli* (middle rows) or *P. aeruginosa* (bottom rows) were incubated on flat control (C, left column), NS (middle column) or NN (right column) surfaces for 3 h or 24 h. Following LIVE/DEAD stain, relative proportions of viable (green) or damaged (red/orange) bacteria were visualised and quantified. Scale bars, 40 *μ*m.

Qualitative evidence from LIVE/DEAD assays indicated significant differences in bacterial growth and viability between *S. aureus, E. coli* and *P. aeruginosa* when bound to the three surface types (Fig. 4). For *S. aureus*, after 3 h of incubation, fewer and a higher proportion of dead/damaged cells was seen on the NN surface than on the control or NS surfaces (Fig. 4 top rows). After 24 h, numbers of cells on all 3 surfaces had increased relative to 3 h. Highest levels of viable cells were seen on the control surface. Dead/damaged cells were visible on both the NS and NN surfaces but while for the NS surface, overall proportions did not seem to change significantly from 3 to 24 h, a higher proportion of dead/ damaged cells was present on the NN surface after 24 h (Fig. 4 top rows). For *E. coli*, lower numbers of bacteria were bound to the NS and NN surfaces after both 3 h and 24 h compared to flat control (Fig. 4 middle rows). As for *S. aureus*, dead/damaged cells were present on both nanostructured surfaces but overall proportions were higher on the NN surface compared to the NS surface (Fig. 4 middle rows). A similar hierarchy of antibacterial effects across the 3 surfaces was seen for

*P. aeruginosa*, which seemed to be the most susceptible to the damaging effects of the NS and NN of the bacterial species tested. In this case, however, overall numbers of bound cells were similar across the 3 surface types (Fig. 4 bottom rows). SEM corroborated the LIVE/DEAD results, revealing a significant reduction in cell populations on NS and NN surfaces compared to controls after 24 h of incubation against all tested strains (Suppl. Fig. S3).

### 3.4 Bacterial response to nanostructures on 3D Ti substrate

**Fig. 5:**
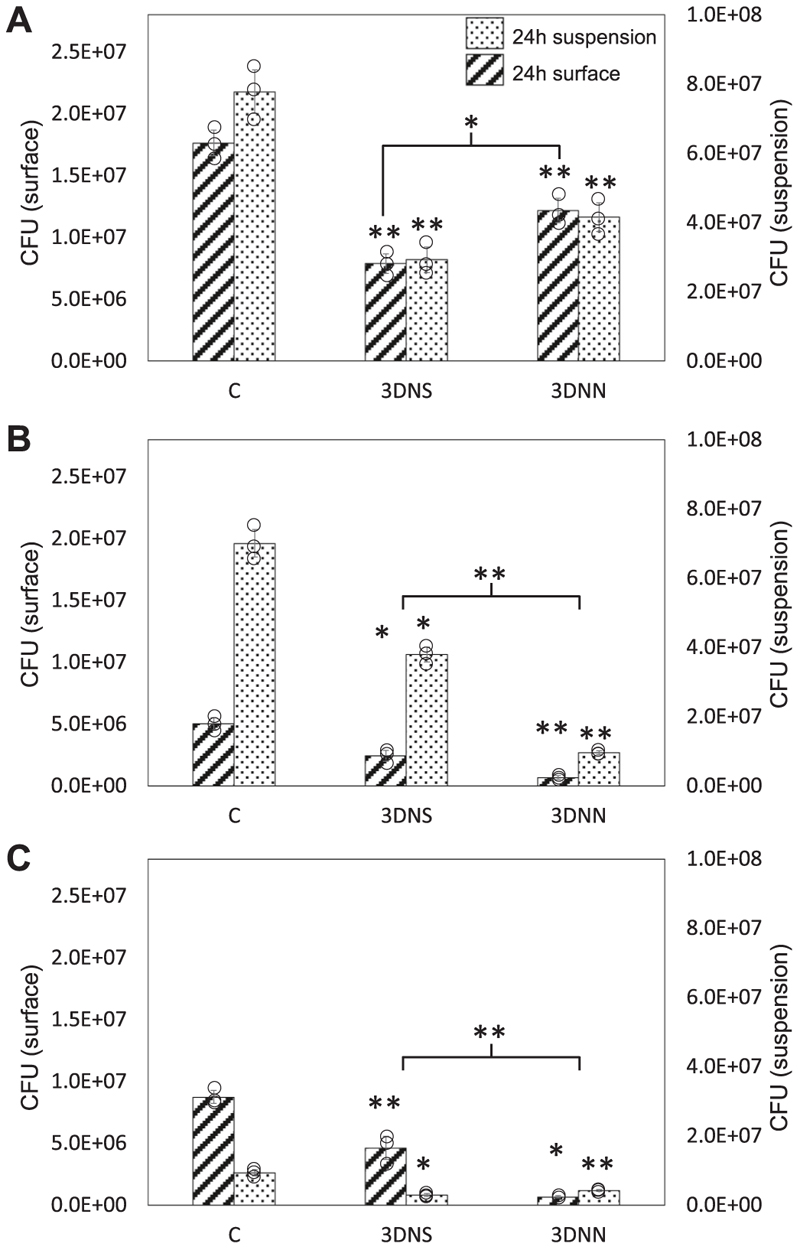
Metabolic activity of bacteria on the surface and suspension of 3D cpTi substrate as measured by BTG assay after 24 h incubation. (a) *S. aureus*, (b) *E. coli*, and (c) *P. aeruginosa*. Data are presented as mean ± SD. *P < 0.05 and **P < 0.01 compared to control and other surfaces, as determined by one-way ANOVA with Tukey HSD post-hoc test; *n* = 3.

BacTiter-Glo assay was also used to assess the antibacterial efficacy of nanostructures on the 3D substrate over 24 h against *S. aureus, E. coli* and *P. aeruginosa*. The same bacterial inoculum was used as for the 2D substrate in a final volume of 400 μl, which was sufficient to immerse the sample. For *S. aureus*, levels of metabolic activity were significantly reduced for both nanostructured surfaces relative to flat control, and both for cells bound directly to the surface and in the surrounding suspension (Fig. 5A). For bound cells, the antibacterial effects of the 3DNS were more pronounced than those of the 3DNN, which had not been seen for the 2D substrate. For both Gram-negative bacterial species, again, levels of metabolic activity were significantly reduced for both nanostructured surfaces relative to flat control but in this instance, the antibacterial effects of the 3DNN were greater than those of the 3DNS (Fig. 5 B,C and Supplementary Fig. S4).

### 3.5 Bacteria/stromal cell co-culture

**Fig. 6:**
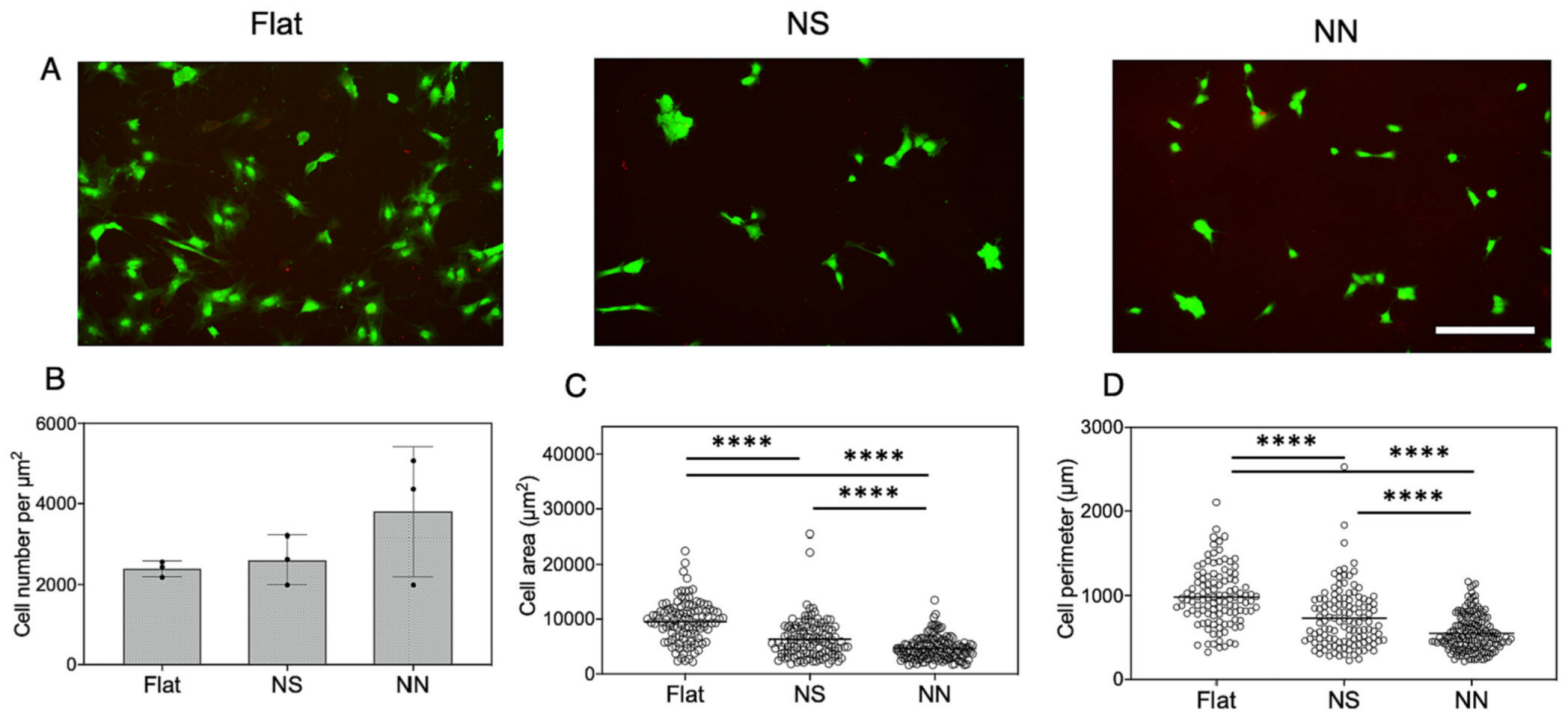
Fluorescence microscopy analysis of hMSC response to 2D nanostructured titanium surfaces after 24 h. (A) Representative LIVE/DEAD stained images showing viable hMSCs on flat, nanospike (NS), and nanonetwork (NN) surfaces. Scale bar is 50 μm. Quantification of (B) hMSC cell numbers, (C) cell area and (D) cell perimeter was performed using Image J software. A threshold was applied, and then particle analysis was performed on the images.

First, the ability of human mesenchymal stromal cells (hMSCs) to adhere and proliferate on NS and NN was assessed over 24 h (Fig. 6A). hMSCs attached to the nanostructured surfaces, although with a less spread conformation than seen on the flat controls. Furthermore, although the numbers of cells present on the surfaces were not significantly different, the cell area and perimeter were significantly lower for hMSCs on the NS or NN surfaces compared to flat control, with the NN surface having the greatest effect on cell morphology (Fig. 6B).

**Fig. 7:**
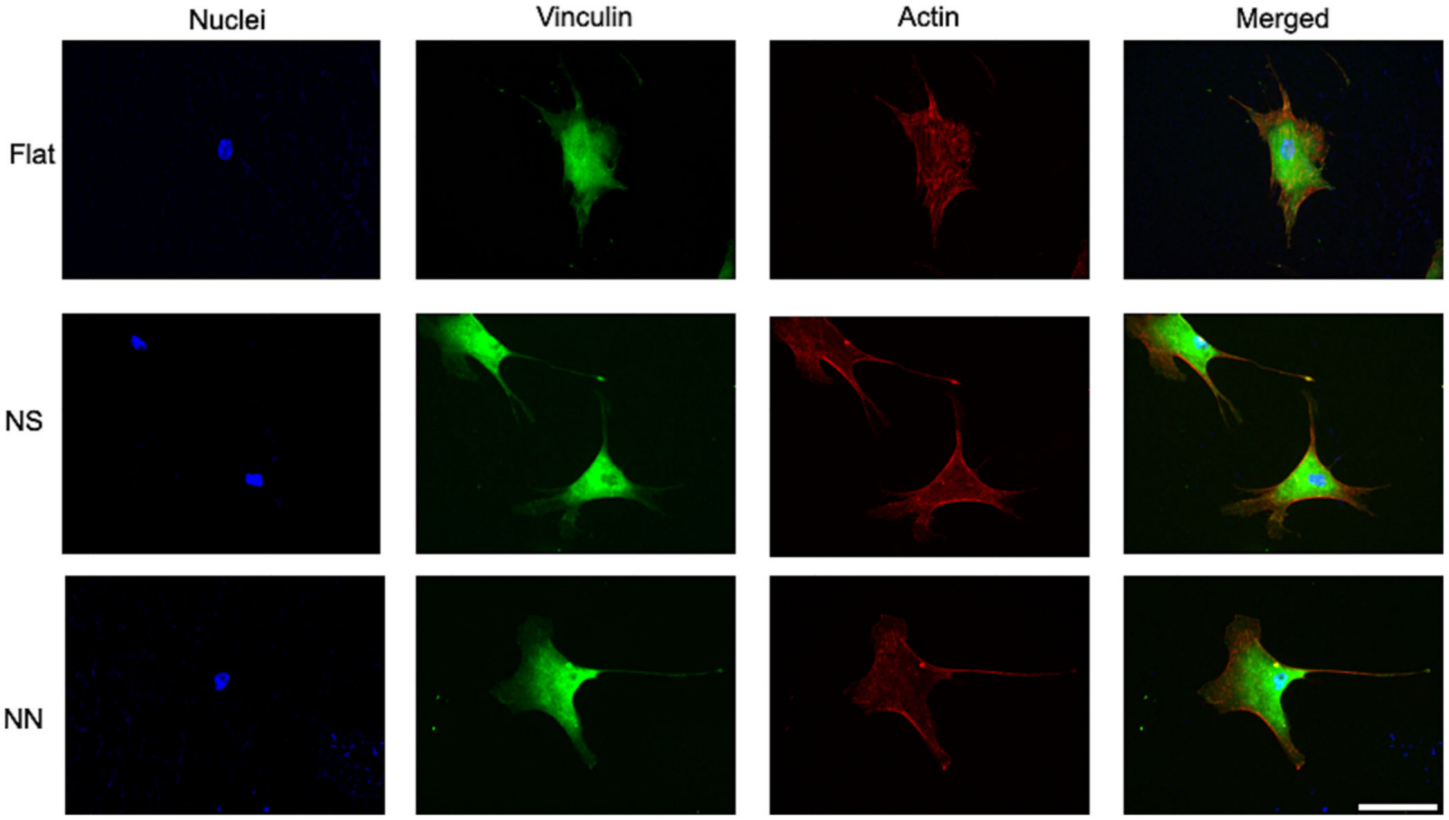
Representative images of the hMSC cytoskeleton following 24 h incubation on 2D cpTi surfaces. hMSCs were incubated for 24 h on flat control, NS or NN 2D cpTi surfaces followed by immunofluorescent staining of vinculin adhesions (green), actin (red) and cell nuclei (blue). Scale bar, 50 μm.

hMSC attachment to the nanostructured Ti surfaces is crucial for possible clinical translation, as this is the first interaction that drives implant osseointegration [17,18]. Therefore, staining of the adhesion-associated protein vinculin was performed, as well as actin, to visualize the response of the cell cytoskeleton to the nanostructures (Fig. 7). This confirmed the establishment of focal adhesions on all three surfaces. However, unlike for the flat control surface, cells seeded on the nanostructured surfaces showed a small body and elongated filaments. This contributed to the smaller area and perimeter of the hMSCs recorded for the two nanostructured surfaces (Fig. 6C,D).

**Fig. 8:**
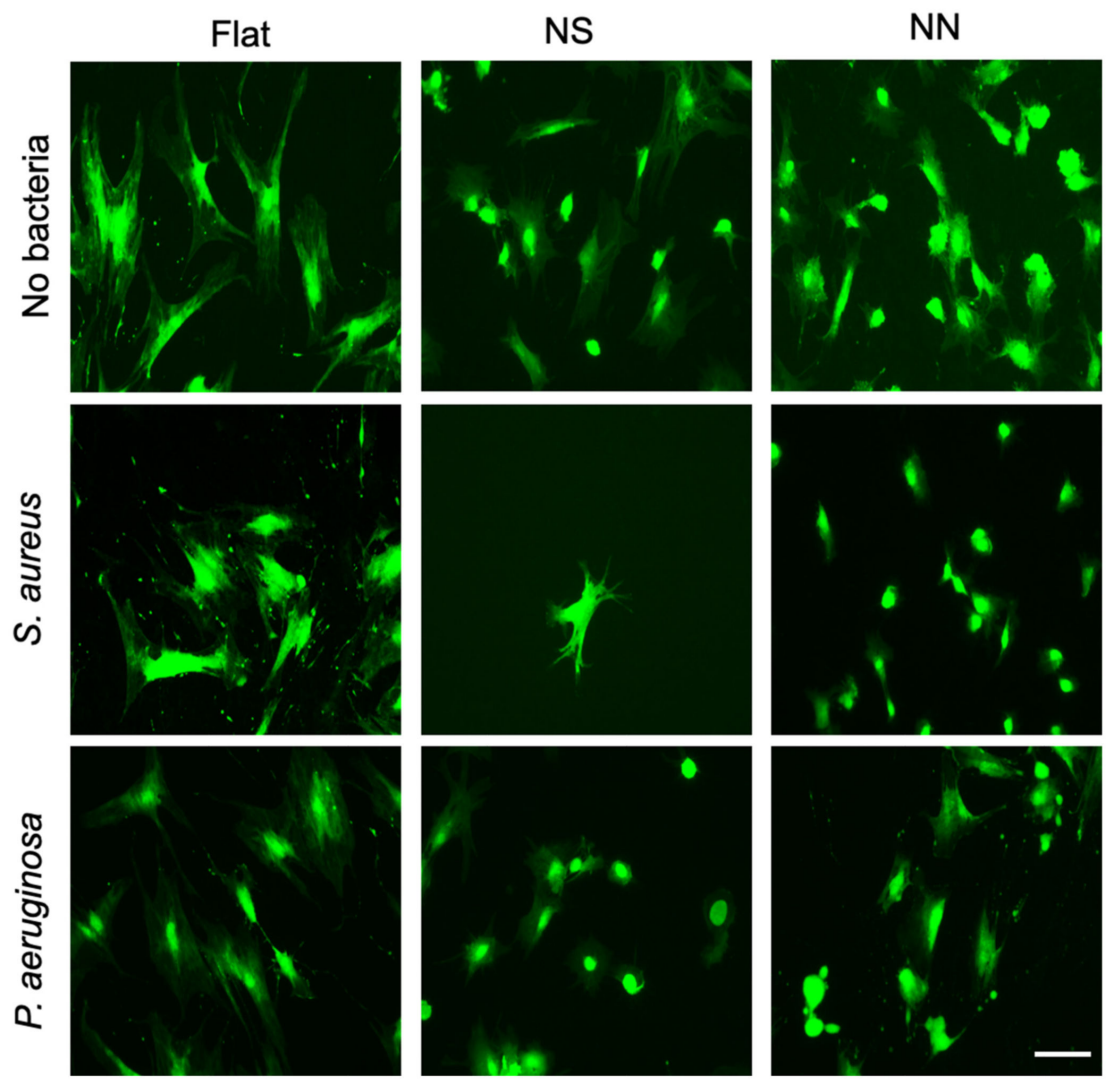
Representative images of hMSCs after 24 h co-culture on 2D cpTi surfaces. hMSCs were incubated for 24 h on flat control, NS or NN 2D cpTi surfaces, followed by incubation for a further 24 h with media alone (top row, control), *S. aureus* (middle row) or *P. aeruginosa* (bottom row). hMSCs were stained with LIVE/DEAD and imaged using a fluorescence microscope. Scale bar, 100 μm.

**Fig. 9:**
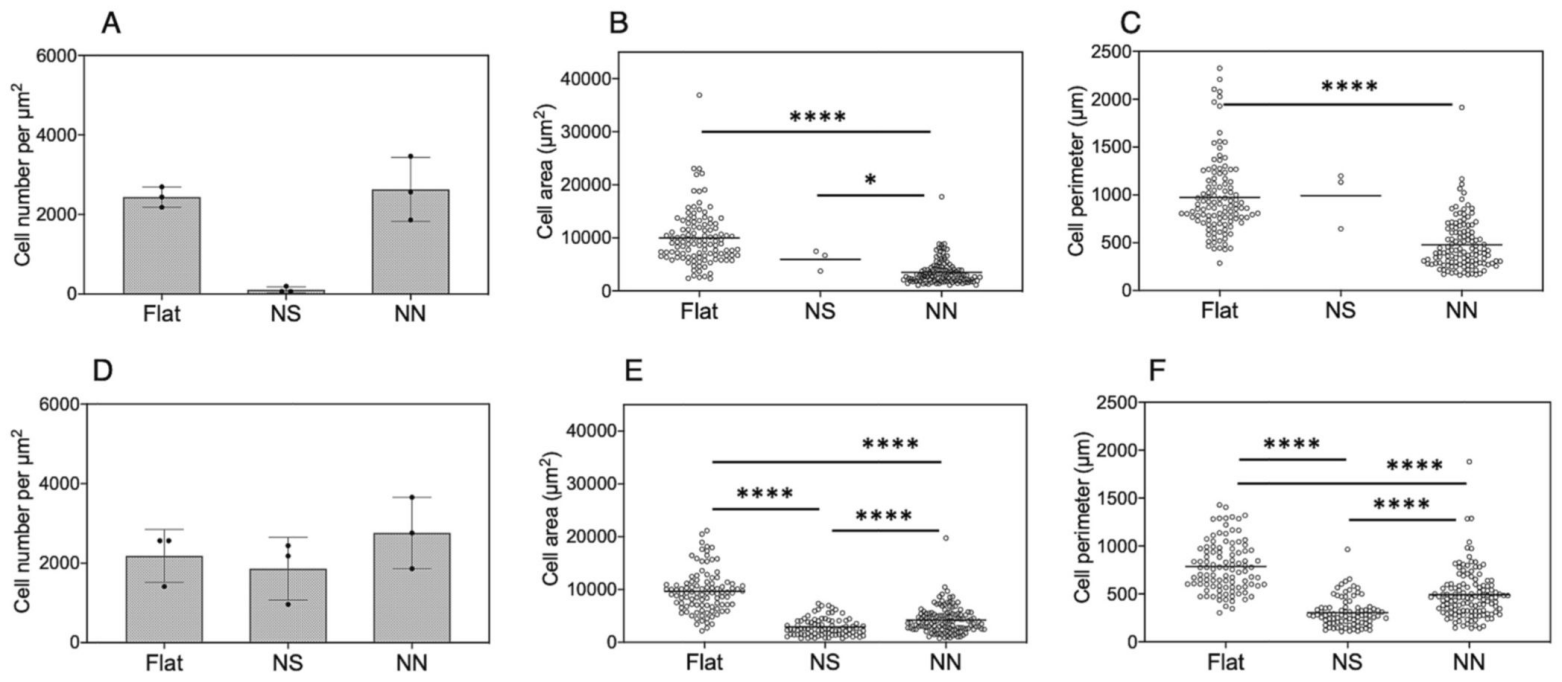
Image quantification of hMSCs following 24 h co-culture on 2D cpTi surfaces. hMSCs were incubated for 24 h on flat control, NS or NN 2D cpTi surfaces, followed by incubation for a further 24 h with *S. aureus* (A-C) or *P. aeruginosa* (D–F). hMSCs were stained with LIVE/DEAD and imaged using a fluorescence microscope. Cell number (A,D), area (B,E), and perimeter (C,F) were quantified using Image J software. A threshold was applied, and then particle analysis was performed on the images. * P < 0.05, **** P < 0.001.

Next, a controlled co-culture scenario was modelled in which hMSCs were seeded onto the 2D cpTi surfaces overnight, followed by inoculation with 10^3^ CFU of either *P. aeruginosa* or *S. aureus* and a further 24 h incubation. LIVE/DEAD stain was performed (Fig. 8) to enable quantification of cell number, area, and perimeter (Fig. 9; Suppl. Fig. S4). Notably, the viability of the hMSCs did not decrease significantly after 24 h of bacterial exposure (Fig. 9 A,D). Again, the hMSC area and perimeter were significantly lower for hMSCs on the NS or NN surfaces compared to flat control in the presence of either bacterial species (Fig. 9 B,C,E,F). However, in the presence of *S. aureus*, NN had the greatest effect (Fig. 9 B,C) whereas this was reversed for *P. aeruginosa* (Fig. 9 E,F).

**Fig. 10:**
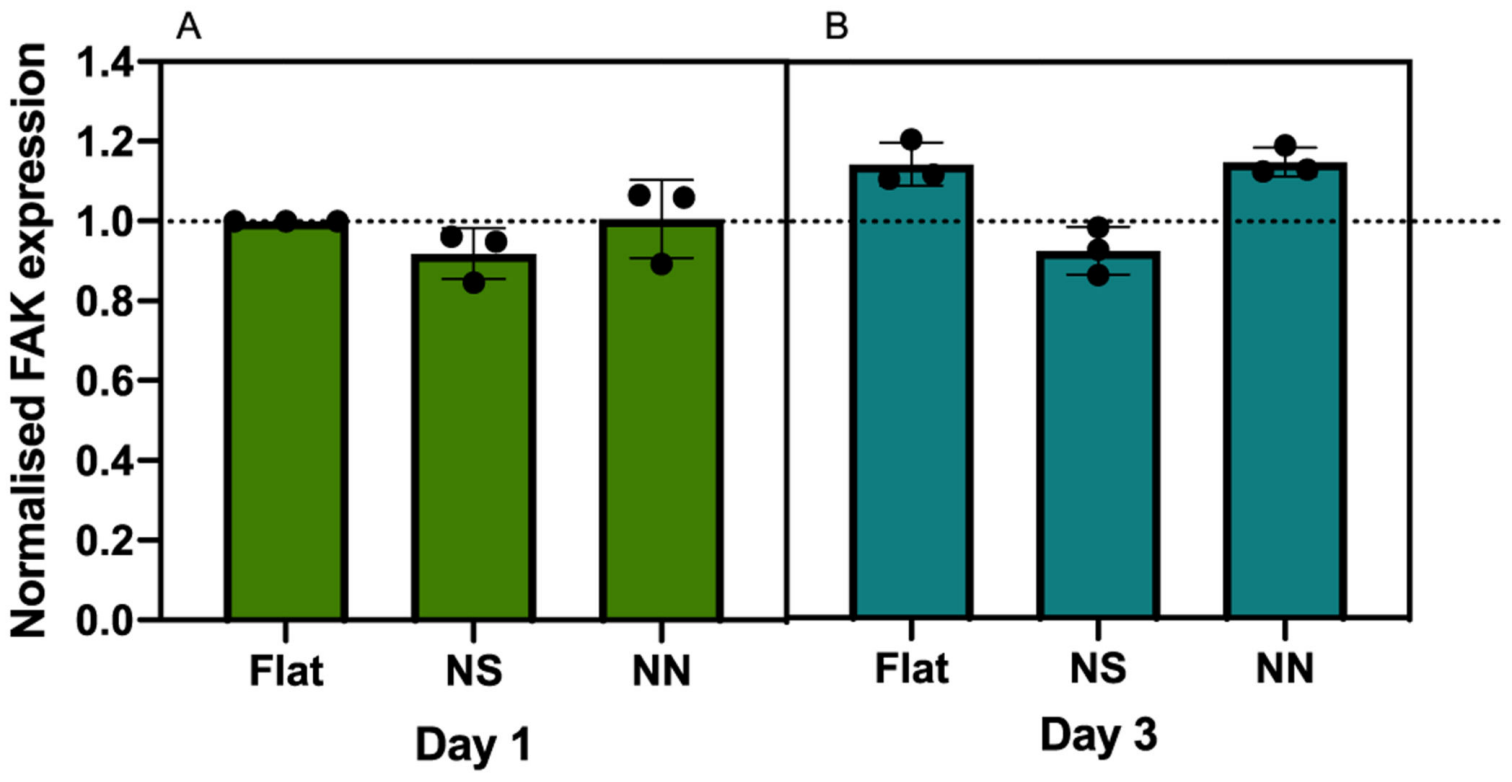
Quantification of focal adhesion kinase in hMSCs following 24 h co-culture on 2D cpTi surfaces. hMSCs were incubated for 24 h on flat control, NS or NN 2D cpTi surfaces, followed by incubation for a further 24 h with *S. aureus*. On day 3, samples were fixed and stained for focal adhesion kinase and the respective phosphorylated conjugate. Data were normalised to the flat control from day 1. Expression levels were compared using an unpaired non parametric *t*-test.

Quantification of focal adhesion kinase (FAK) after co-culturing hMSCs with *S. aureus* on the 2D cpTi surfaces was performed using in-cell Western analysis and taking the ratio of phFAK to total FAK to account for active FAK (Fig. 10). No significant difference was seen in FAK expression across the three surface types; however, there was a trend for the hMSCs to recuperate from day 1 to day 3 after bacterial exposure, especially for hMSCs seeded onto the NN surface.

**Fig. 11:**
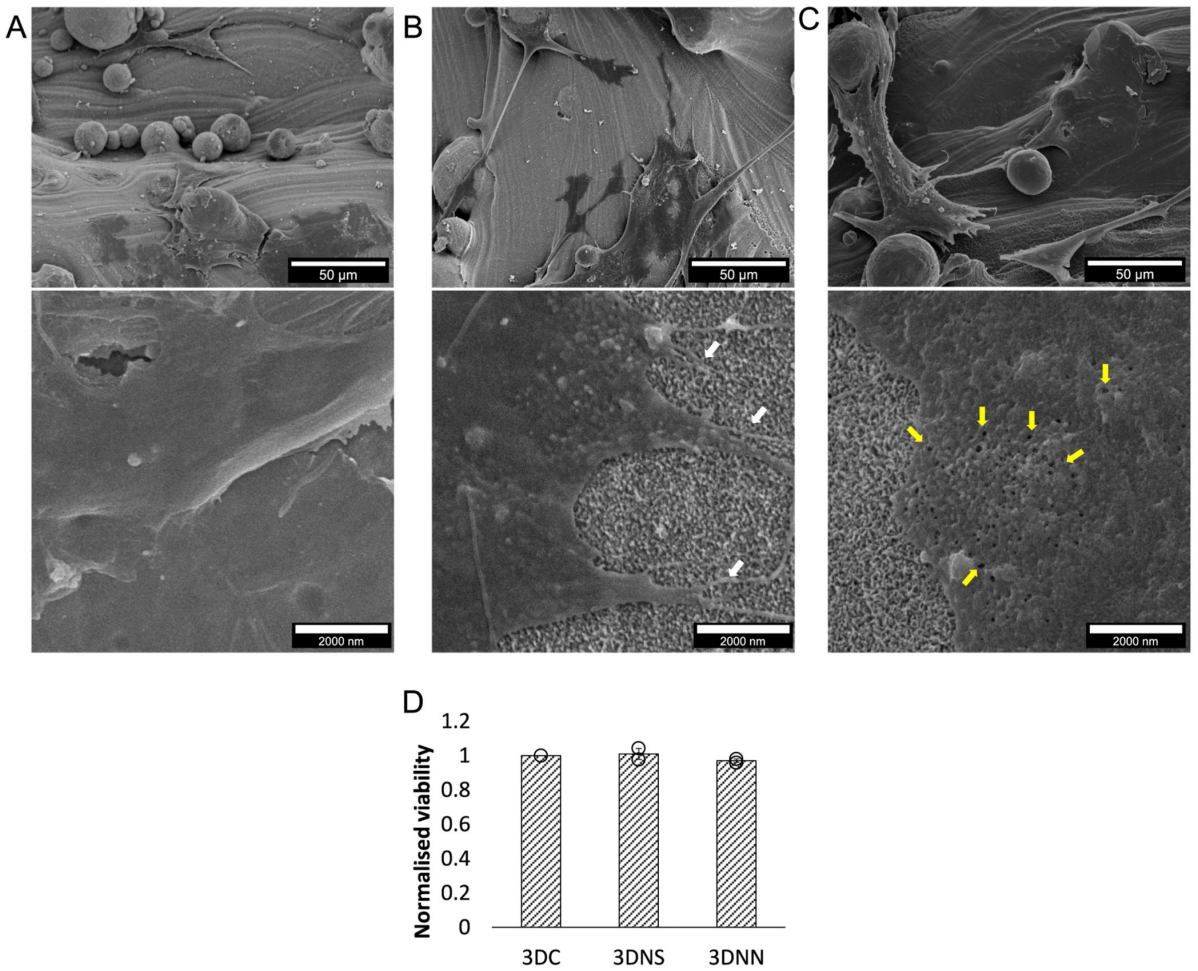
Response of hMSCs to 3D cpTi substrates after 24 h. hMSCs were cultured for 24 h on flat control (3 DC), 3DNS or 3DNN cpTi surfaces and observed via SEM (A-C), where the top panel represents low magnification. In the bottom panel high magnification images, white arrows on the 3DNS image indicate a significantly higher number of filopodia extending to the surface, a phenomenon not observed on 3 DC and 3DNN. Meanwhile, yellow arrows on the 3DNS surface point to areas where hMSCs seem to integrate within the nanonetwork, revealing visible “pockets”. Cell viability was measured by Alamar blue staining (D).

Finally, the response of hMSCs to the 3D cpTi substrate was investigated. Viable hMSCs were seen on all 3 surface types after 24 h, with similar levels of metabolically active cells present (Fig. 11). A typical adhesion profile was seen for the hMSCs on the 3 DC substrate. While hMSCs on the 3DNS exhibited elongated appendages (white arrows). By contrast, the hMSCs appeared to form small “pockets” (yellow arrows) when adhering to the 3DNN.

## 4 Discussion

### 4.1 Nanospike vs nanonetwork: potential antibacterial mechanism

There were no significant differences between the antibacterial effects of NS and NN on 2D substrates but when translated onto 3D substrates, differential bacterial responses were observed (Fig. 5). *S. aureus* was more susceptible to NS, while Gram-negative bacteria were more susceptible to NN. This could be due to the differences in nanostructure density between NS (43 nanostructures *μ*m^-2^) and NN (6 nanostructures *μ*m^-2^). Recently, we showed that *S. aureus* was more susceptible to sharp and dense PET nanopillars compared to blunt and wide nanopillars [19]. High-density nanopillars had higher contact points with the bacteria and were anticipated to inflict more damage on the cells due to induction of a stress response and membrane deformation [8,13,19]. It is predicted that a similar mechanism underpins the susceptibility of *S. aureus* to 3DNS seen here. However, due to the low density of nanostructures found on 3DNN, the mechanobactericidal effects of the NN were limited against *S. aureus*.

Our NN surface consisted of elongated nanowires, where some of the long nanospikes intertwined and formed a dome structure. A cross-section of the NN surface revealed that within the intertwined structure, smaller nanospikes were present (Suppl. Fig. S1). This unique feature served to increase the total surface area of the NN surface, despite having a lower density of nanostructures compared to the NS surface. It is also important to note that the total surface area for NN could be higher than by AFM (Fig. 1) due to AFM tip/sample convolution [20]. This could explain the higher antibacterial activity of NN against Gram-negative bacteria reported here, which differs from previous studies that have shown higher-density nanostructures to have a better antibacterial efficacy towards Gram-negative and Gram-positive bacteria compared to low-density nanostructures [19,21]. It is predicted that the intrinsic pressure is higher on the NN than on the NS surface because the bacterial cell is maximising its adhesion to the incontact and surrounding nanostructures (within the intertwined nano-spikes) [19].

Differences between the antibacterial efficacies of the nanostructures on 2D compared to 3D substrates could be attributed to the gyroid lattice structures of the 3D substrate. It is possible that the differential fluid pressure found around the gyroid lattice structure affected the swimming dynamics of the cells during attachment to the surface, together with the flow velocity in the 3D substrate. For motile bacteria like *E. coli* and *P. aeruginosa*, this could have made their swimming more chaotic and subsequently increased tumbling and contact with the nano-structures during the early stage of adhesion [22,23].

In addition to the bactericidal effects of the nanostructures, the ability of NS and NN architecture to inhibit bacterial growth is also likely to be multifactorial [21,24]. Factors that have been implicated in impairing bacterial replication include nanostructure-mediated induction of intracellular reactive oxygen species (ROS) [8] and physical impedance of the bacterial cells when trapped between nanostructures [8,19,26].

Potential novel material for antibacterial and biocompatible orthopaedic implants.

Several recent studies [27] have reported successful fabrication of nanostructures on mainly 3D printed titanium alloy substrates such as Ti6Al4V, using techniques like alkaline hydrothermal treatment [16,28], anodisation [29] and acid etching [30]. However, pure titanium remains one of the most clinically important materials for orthopaedic and dental implants, and a key gap persists in directly generating comparable nanostructures on matched 2D and 3D cpTi substrate using a scalable nanofabrication technique.

The results presented in this study demonstrate the potential of nanostructured titanium surfaces, especially the NN topology, as effective antibacterial and biocompatible implant materials. Both NS and NN structures fabricated on both 2D and 3D titanium substrates were shown to significantly reduce metabolic activity of Gram-positive and Gram-negative bacteria (Fig. 3, Fig. 5). This antibacterial effect is likely due to the nanostructures disrupting adhesion and proliferation of bacterial cells at the surface [19]. Meanwhile, hMSCs remained viable and were able to adhere, spread, and migrate across all nanostructured surfaces, even in the presence of bacteria (Fig. 8, Fig. 9).

It was found that the presence of *S. aureus* had the most detrimental effect on the viability of hMSCs when incubated with the NS surface (Fig. 8). Such effects have been reported previously for MSCs on a flat surface (i.e. well plate) in the presence of *S. aureus*, and it has been suggested that *S. aureus* may induce MSC apoptosis through the release of α-toxins and Panton-Valentine leukocidin [31–33]. A similar mechanism may explain the data reported here for the NS surface. Furthermore, the different levels of hMSC viability across the NS and NN surfaces may, in turn, reflect differences in *S. aureus* on the two surfaces. As evidenced by LIVE/DEAD staining (Fig. 4), a higher proportion of *S. aureus* cells exhibited signs of a compromised cell membrane on the NN surface compared to the NS surface, such conditions may limit the release of toxins by *S. aureus*. This will be an interesting hypothesis to test in future studies.

Interestingly, SEM images (Fig. 11), revealed distinct responses of hMSCs to the 3 DC, 3DNN and 3DNS surfaces. Notably, the cells on 3 DC exhibited a typical adhesion profile, as anticipated. However, on 3DNS, a significantly higher proportion of hMSCs displayed substantial extension with pronounced appendages than seen for other surfaces. In the case of 3DNN, there appeared to be small “pockets”, which indicates that the cells were intricately embedded within the nanonetwork. These results corroborate with our previous findings where we reported enhanced hMSC adhesion, increase osteopontin expression, and reduced biofilm formation on 3D printed titanium alloy substrates coated with nanowires and fibronectin [16]. This highlights a key advantage of the nanostructure approach, where the 3D porous architecture allows migration and proliferation of larger eukaryotic cells, while bacteria are restricted to the surface. Such differential responses could help tip the race for the surface in favour of human cells over bacteria. While the results from this study do not show direct evidence that 3D nano-structured surfaces can improve integration by winning the race to the surface against bacteria, this remains an important hypothesis to investigate further. Future studies could employ a co-culture model of hMSCs and bacteria to assess the competitive adhesion and proliferation of these cells on 2D and 3D substrates with promising nanostructure designs.

The 3D printed titanium substrate possessing a gyroid structure with 60 % porosity demonstrated mechanical and permeability properties closely matching cortical bone [12]. Importantly, the successful generation of uniform nanostructures on 3D printed cpTi substrates via alkaline etching represents a direct translation of this technique from 2D to 3D substrates using an identical material and approach. Previous study showed enhanced antibacterial efficacy of cpTi nanospikes with fibronectin coating. However, they utilised a different 3D printed titanium alloy substrate. Our work is the first demonstration of generating comparable nanostructures on both 2D and 3D cpTi to elicit similar bacterial responses. Together, these results highlight the promise of nanoengineered titanium surfaces, particularly the interconnected NN topography, to simultaneously resist bacterial adhesion while supporting human cell functions for improved orthopaedic implant integration and prevention of infections.

Considering the promising in vitro results for the nanospikes and nanonetwork on both 2D and 3D substrates, additional experimentation will be required before potential translation of this work to an in vivo model. For instance, flow cell experiments modelling physiological fluid shear will allow the assessment of bacterial adhesion and biofilm formation on the nanostructures over extended periods of time under clinically-relevant dynamic conditions [34]. Understanding nano-structure stability and retention of antibacterial function after exposure to harsh mechanical forces that mimic the implant surgical procedures is also critical. Such additional in vitro optimization and testing would facilitate identification of the most promising nanostructured implant designs for subsequent in vivo assessment.

Applying nanostructure fabrication using an alkaline etching technique or other similarly scalable nanofabrication approach to other medically-relevant materials (e.g. Ti6Al4V or Ti6Al7Nb alloy) and to complex device/scaffold geometries would provide important preclinical validation. Recent work by Bright et al. demonstrated the feasibility of using an alkaline hydrothermal method to fabricate two distinct nanostructures on Ti6Al4V 2D substrates [35]. The high-density nano-structures (75 nanostructures/*μ*m^2^) that were etched with NaOH were found to be more effective against Gram-negative bacteria, while the low-density nanostructures (8 nanostructures/*μ*m^2^) etched with KOH were more effective against Gram-positive bacteria. Importantly, both\ nanostructured Ti6Al4V surfaces maintained comparable levels of human dermal fibroblast viability and growth to flat controls.

Overall, this study provides strong foundational insights into the biological performance of nanostructured titanium surfaces fabricated through straightforward alkali-heat treatments. Both the NS and NN topographies present readily scalable approaches to confer antibacterial properties while maintaining biocompatibility on promising 3D printed metallic orthopaedic implants. Further in vivo assessments will be important next steps to fully evaluate their potential to improve clinical outcomes.

## 5 Conclusion

In conclusion, our study investigated the antibacterial properties of two distinct nanostructures, NS and NN, on both 2D and 3D substrates. Both nanostructures exhibited antibacterial activity, but efficacy varied based on bacterial strain and substrate geometry likely due to nano-structure density and surface area differences. Specifically, *S. aureus* was found to be more susceptible to 3DNS, while Gram-negative bacteria showed higher susceptibility to 3DNN. This was likely due to variations in nanostructure density and surface area between the substrates especially with our NN topography which have low density high surface area architecture. Moreover, hMSCs were able to firmly attach to NS and NN surfaces in both 2D and 3D at levels that were comparable to the flat surface and remain metabolically active, even in the presence of bacteria. This suggests that both nanostructured surfaces may be promising materials for biocompatible orthopaedic implants that can compete against bacteria for the surface. This study contributes to the growing body of research on the design and development of nanomaterials for antibacterial and biomedical applications, and highlights the importance of considering substrate geometry and bacterial species during the design process.

## Supplementary Material

Supplementary data to this article can be found online at https://doi.org/10.1016/j.bioadv.2024.213766.

Supplementary Material

## Data Availability

Data will be made available on request.
