# Supplementary material for: Nanotextured titanium inhibits bacterial activity and supports cell growth on 2D and 3D substrate: A co-culture study
Source: Biomater Adv. Author manuscript; Available in PMC 2025 Apr 2. (PMC7617543; doi:10.1016/j.bioadv.2024.213766)
Supplement: Supplementary Material [file EMS204120-supplement-Supplementary_Material.docx]

Nanotextured Titanium Inhibits Bacterial Activity and Supports Cell Growth on 2D and 3D substrate: A Co-Culture Study

Mohd I. Ishak^1,2^, Rosalia Cuahtecontzi Delint^3^, Xiayi Liu^1^, Wei Xu^4^, Penelope M. Tsimbouri^3^, Angela H. Nobbs^1^, Matthew J. Dalby^3^, and Bo Su^1*^

1. Bristol Dental School, University of Bristol, Lower Maudlin Street, Bristol, BS1 2LY, UK

2. School of Chemistry, University of Bristol, Cantock's Close, Bristol, BS8 1TS, UK

3. Centre for the Cellular Microenvironment, School of Biomedical Sciences, University of Glasgow, Glasgow, G12 8QQ, United Kingdom

4. National Engineering Research Center for Advanced Rolling and Intelligent Manufacturing, Institute of Engineering Technology, University of Science and Technology Beijing, Beijing 100083, China

Keywords: 2D and 3D substrate, co-culture, nanostructures, antibacterial, biocompatible

## Supplementary Tables

Table S1. Bacterial strains used and relevant characteristics.

| Strain ID | Bacterial Strain | Morphology | Gram Identity | Motility | Reference / Source |
| --- | --- | --- | --- | --- | --- |
| UB 776 | *Escherichia coli* DH5α | Bacillus | Gram negative | Motile | ThermoFisher Scientific |
| ATCC 27853 | *P. aeruginosa* (Schroeter) Migula | Bacillus | Gram negative | Motile | ATCC |
| UB 1621 | *Staphylococcus aureus* Newman | Coccus | Gram positive | Non-motile | [1] |

**Supplementary Figures**


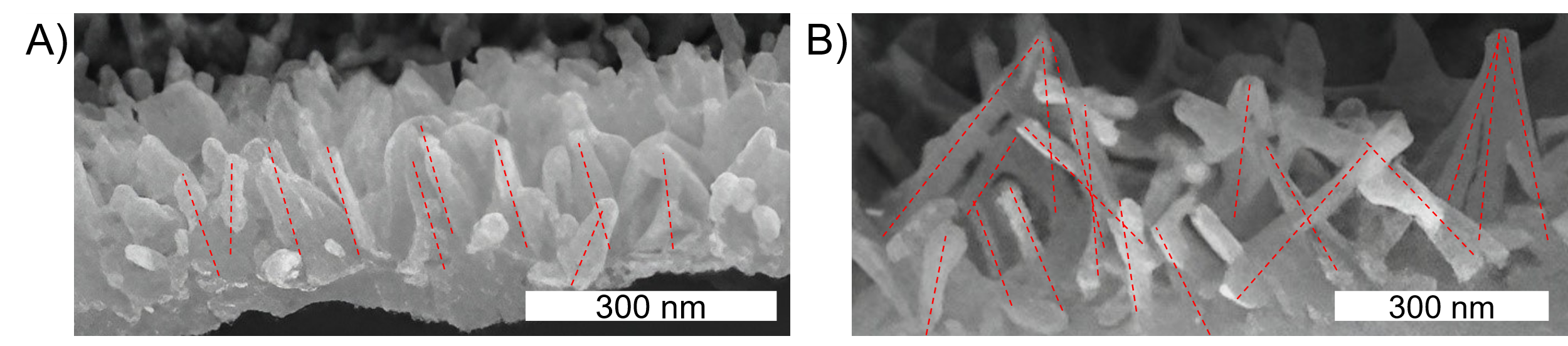


Supplementary Figure S1. Cross section SEM image of (A) NS and (B) NN. Most structures on the NS surface were parallel to each other while some NS grew at an angle. As the cpTi was etched for longer, some of the NS grew taller and formed a NN, which consisted of interconnected long NS with short NS around it. This unique property gave NN a low density, high surface area structure compared to NS.


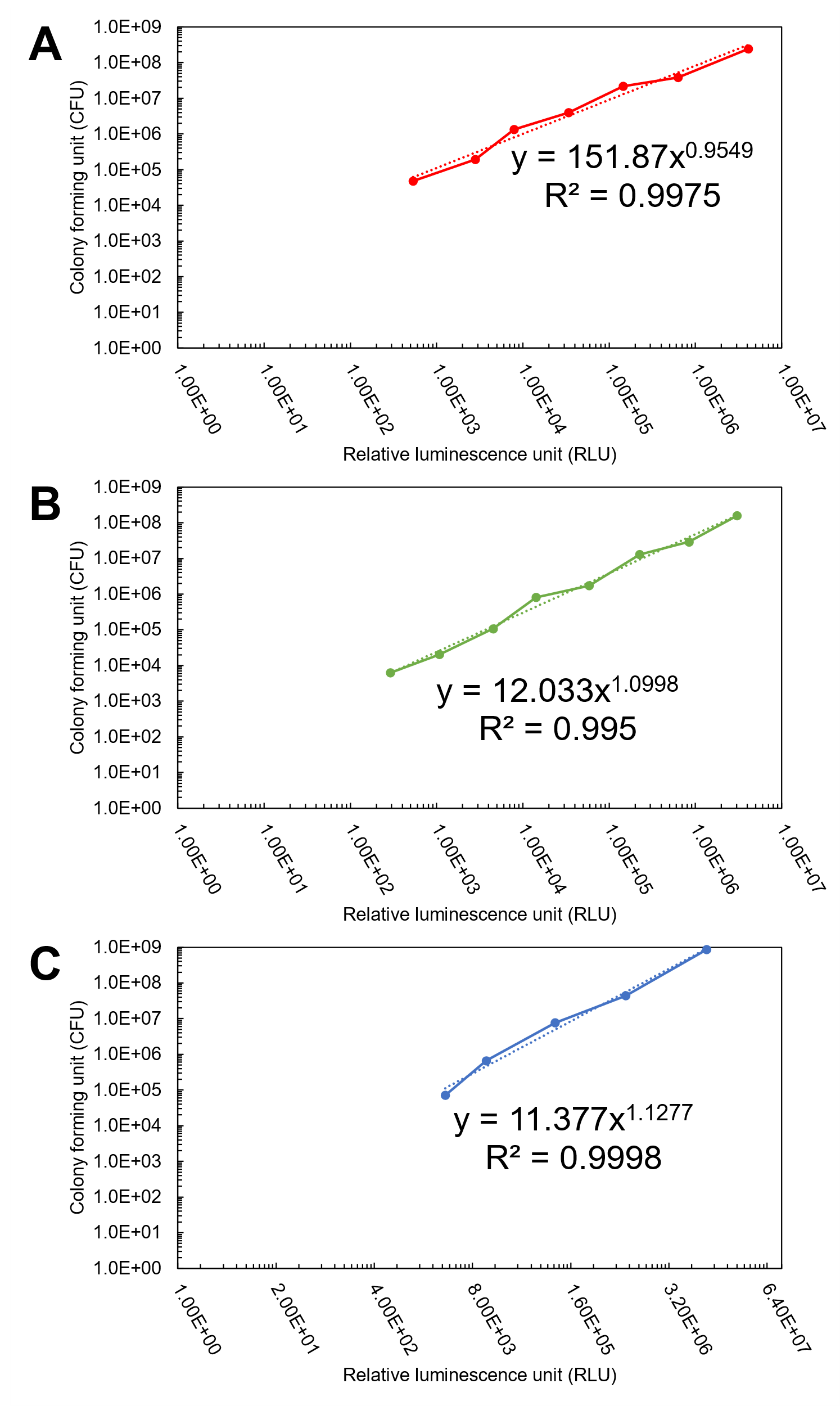


Supplementary Figure S2. Standard curves for correlation between luminescence signal of BTG reagent with CFU. Bacterial viability (CFU) on cpTi surfaces was quantified by converting raw luminescence signal (RLU) from BTG experiments using the line equation for (A) *S. aureus,* (B) *E. coli*, and (C) *P. aeruginosa.* (A) and (B) were reproduced with permission from [2].


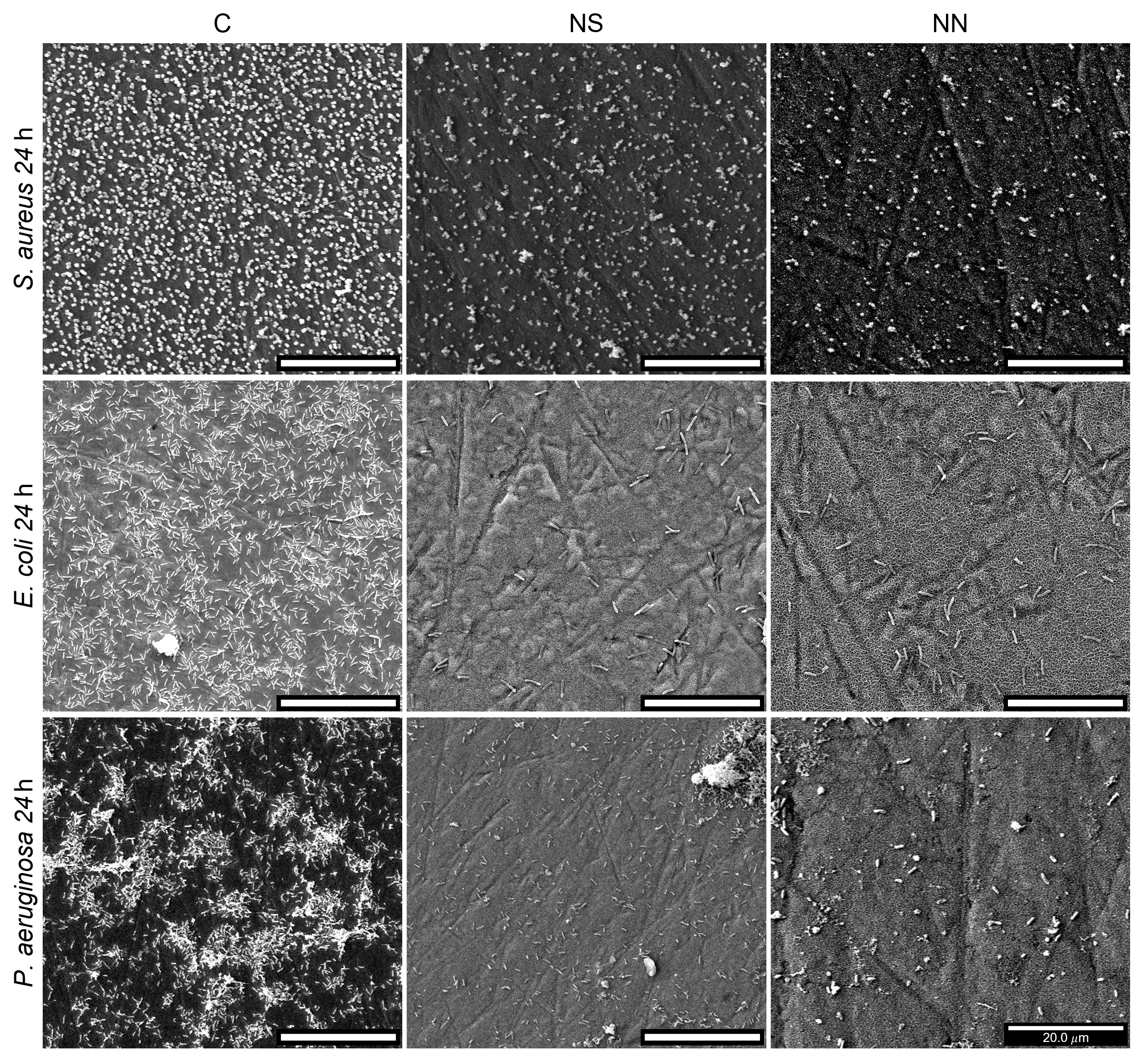


Supplementary Figure S3. Bacterial response to 2D nanostructured surface. Representative scanning electron micrograph images of *S.* aureus, *E. coli,* and *P. aeruginosa* on flat control, NS, and NN surfaces after 24 hours of static incubation. Scale bar is 20 $\mu$m.

Supplementary Figure S4. *S. aureus* response to nanostructures on 3D substrate. Representative SEM images of *S. aureus* on (A) 3DC, (B) 3DNS, and (C) 3DNN substrates after 24 hours of static incubation. Top panel, low magnification images; bottom panel, high magnification images.


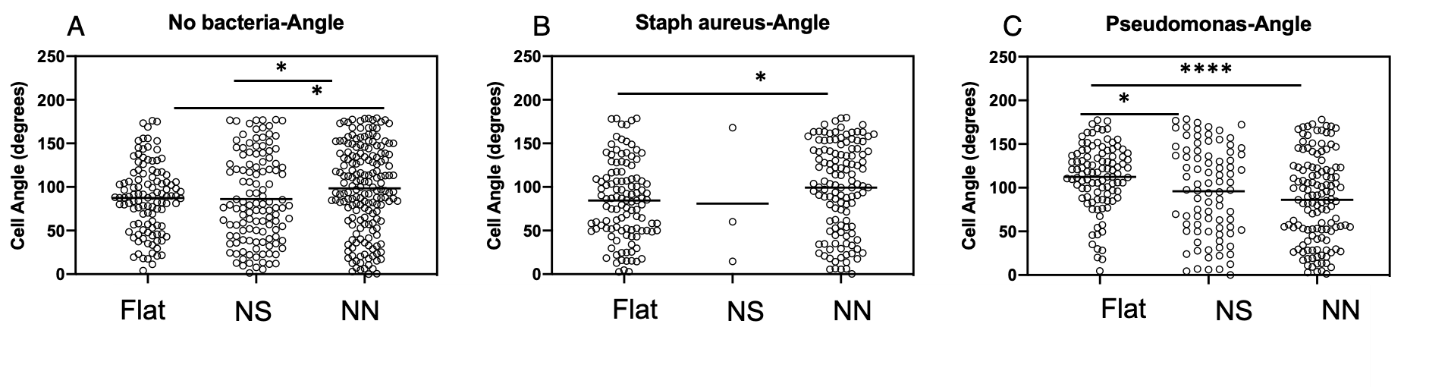
Supplementary Figure S5. Quantification of cell angle after bacteria co-culture overnight. (A) Ti surfaces without bacteria, (B) hMSCs incubated with *S. aureus*, and (C) hMSCs incubated with *P. aeruginosa* for 24 hours. Live/Dead staining was performed, and images were taken using a fluorescence microscope. Angle quantification was performed using Image J.

**REFERENCES**

[1] Duthie, E. S. & Lorenz, L. L. Staphylococcal coagulase: mode of action and antigenicity. Microbiology **6**, 95–107 (1952).

[2] Jenkins, J. *et al.* Antibacterial effects of nanopillar surfaces are mediated by cell impedance, penetration and induction of oxidative stress. Nat. Commun. **11**, 1–14 (2020).
